# T_H_9 cell differentiation, transcriptional control and function in inflammation, autoimmune diseases and cancer

**DOI:** 10.18632/oncotarget.11681

**Published:** 2016-08-29

**Authors:** Yan Li, Qing Yu, Zhengguo Zhang, Jian Wang, Simin Li, Jiangyuan Zhang, Guangwei Liu

**Affiliations:** ^1^ Key Laboratory of Cell Proliferation and Regulation Biology of Ministry of Education, Institute of Cell Biology, College of Life Sciences, Beijing Normal University, Beijing, China; ^2^ Department of Immunology, School of Basic Medical Sciences, Fudan University, Shanghai, China

**Keywords:** T cell differentiation, T_H_9, allergic airway inflammation, tumor, autoimmune diseases

## Abstract

Naïve CD4^+^T cells differentiate into various T cell subsets depending on the specific cytokine environment. T_H_9 cells are less well-characterized than other T cell subsets, and factors that control their development and function have only recently been identified. It is now clear that T_H_9 cells play critical roles in immune-mediated diseases, including allergic airway, autoimmune and inflammatory bowel diseases, and cancer. Thus, the promotion or suppression of T_H_9 cell differentiation, transcriptional control and function may provide novel treatments for clinical inflammation, autoimmune diseases and tumors.

## INTRODUCTION

**Figure 1:**
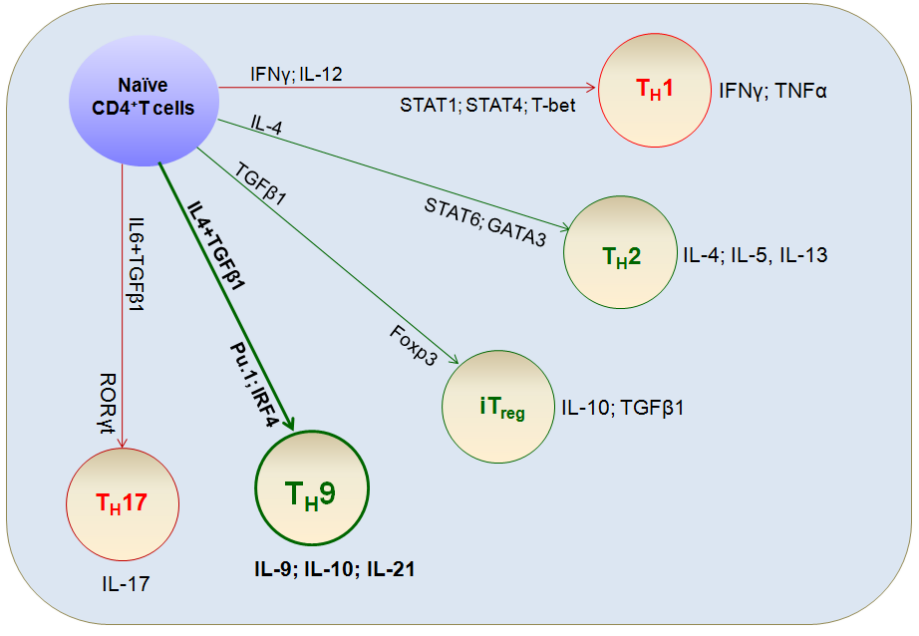
Differentiation of T cell lineages Naïve CD4^+^T cells are activated by T cell receptor (TCR) signaling and differentiate into various T cell lineages depending on the cytokine environment. Prototypical differentiation cytokine sets, corresponding specific transcriptional factors, and functional cytokine effects that regulate T_H_ cell fate and functions (including T_H_9 cells) are shown.

After T cell antigen receptor activation, the fate of naïve T cells is determined to a large extent by the cytokine environment [1–4]. Naïve CD4^+^T cells differentiate into functionally distinct subsets, including IFNγ-producing T_H_1 cells, IL-4-producing T_H_2 cells, IL-17-producing T_H_17 cells, and induced regulatory T cells (iT_reg_); these cells are responsible for different types of T cell immunity and affect immune-mediated responses to disease [5–8] (Figure 1). Among these T cell subtypes, IL-9-producing T_H_9 cells were first described in 1994 [9] and defined in 2008 [10, 11]. Initially, T_H_9 cells were thought to be associated with T_H_2 responses and to arise from TGFβ-induced reprogramming of T_H_2 cells [12–14]. However, recent reports have shown that T_H_9 cells are involved in antimicrobial immunity [15, 16], autoimmune disease [17–19], colitis [20, 21], and even anti-tumor immunity [12, 22]. This review summarizes the emerging role of T_H_9 cells in immune-mediated diseases, with a special focus on several recent anti-tumor immunological studies.

## T_H_9 CELL DIFFERENTIATION AND SIGNAL TRANSDUCTION

### T_H_9 cell differentiation

The cytokine milieu plays a crucial role in T cell differentiation. In addition to IL-4 and TGFβ [10, 11], IL-1 [23], IL-2 [24], IL-6 [25], IL-10 [26], IL-21 [27, 28], IL-23 [29], IL-25 [30], IL-33 [31], IFN-α/β [27], and thymic stromal lymphopoietin (TSLP) [32] promote IL-9 production in naïve T cells, while IFN-γ and IL-27 suppress IL-9 production [18]. For example, naïve CD4^+^T cells primed with IL-4 and TGFβ1 secrete IL-9 [9]. Compared to stimulation with TCR alone, the addition of TGFβ1 increased IL-9 production in murine CD4^+^T cells; IL-9 production was further increased by the addition of both TGFβ1 and IL-4, which, by itself, has scant effect [33–36]. A variety of cytokines are now known to affect Th9 cell differentiation and IL-9 production in T cells.T_H_9 cell development requires the integration of multiple signals, and a complex cytokine milieu is required for optimal IL-9 production; the balance of cytokine signals is therefore critical for inducing T_H_9 cell development and differentiation rather than the generation of other T-helper subsets.

### Transcriptional control of T_H_9 cell differentiation

**Figure 2:**
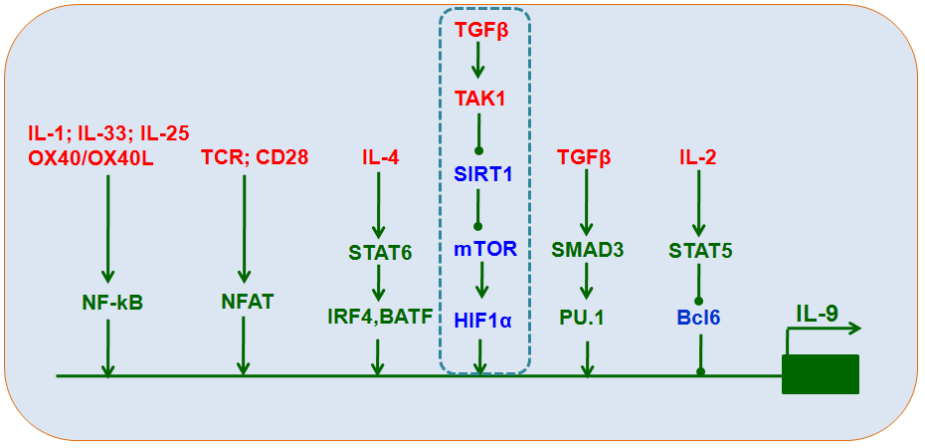
Transcriptional control of T_H_9 cell differentiation IL-4, TGFβ1, IL-2, TCR, and other stimuli induce the expression of downstream transcriptional factors, including STAT6, IRF4, BATF, SMAD3, TAK1-SIRT1-mTOR-HIF1α, PU.1, STAT5, Bcl6, NF-kB, and NFAT, that interact with *Il9* promoters to increase IL-9 expression and secretion.

Certain transcription factors, including STAT [37], PU.1 [21, 38, 39], IRF1 [28], IRF4 [40], NF-kB [41], Bcl6 [42], and the Smad/Notch complex [43], directly interact with the *Il9* gene promoter to increase IL-9 production (Figure 2). Moreover, acetylation and H3K27 trimethylation, a suppressive chromatin modification, are increased at the IL-9 promoter in T_H_9 cells [39, 44], resulting in barely detectable IL-9 production in these cells compared to other CD4^+^T cell lineages.

IL-4 and TGFβ1, and their downstream transcriptional targets, are required for T_H_9 cell differentiation [23, 45]. For example, IL-4-induced activation of STAT6 and the STAT6 target gene GATA3 are both required for T_H_9 differentiation, although GATA3 is more important for T_H_2 differentiation [13, 46]. Upon activation, phosphorylated STAT6 facilitates the transcription of GATA3 and IRF4 [47]. However, modest retrovirus transduction-induced expression of IRF4 and/or GATA3 did not rescue IL-9 secretion in STAT6-deficient CD4^+^T cells, indicating that additional factors are required for the STAT6-dependent transcriptional modulation of T_H_9 differentiation [46]. In addition, GATA3 transcription is activated in a STAT6-independent manner during T_H_9 differentiation. Notch1- and Notch2-deficient T_H_9 cells exhibit decreased IL-9 production; Jagged2 is able to induce IL-9 production in the presence of TGFβ1 alone in these cells, and exogenous IL-4 rescues Notch deficiency [48, 49]. The DNA-binding inhibitor Id3 inhibits IL-9 production in CD4^+^T cells in a GATA3-dependent manner [33]. Deletion of Id3 increases IL-9 production in CD4^+^T cells, indicating that Id3 also inhibits T_H_9 differentiation in an IL-4-GATA3-dependent manner. These data suggest that STAT6 signaling is not absolutely necessary for the induction of T_H_9 differentiation; Notch or Id3-mediated induction of GATA3 is sufficient.

TGFβ is also required for T_H_9 generation. Accordingly, the TGFβ downstream target factor SMAD is critical for T_H_9 cell differentiation. Binding of TGFβ to its receptor activates specific SMAD family members, and TGFβ-activated phosphor-SMAD3 directly binds to the *Il9* locus, the Notch intracellular domain (NICD), and RBP-Jk (recombination signal binding protein for immunoglobulin kappa J region) [10, 43]. In addition, TGFβ1 induces transcriptional factor PU.1 expression and inhibits the expression of T-bet, a T_H_1-specific transcriptional factor, thereby promoting T_H_9 differentiation [21, 39]. PU.1 is expressed specifically in subpopulations of T_H_2 cells with low IL-4 expression. PU.1-deficient T cells produce less IL-9, and ectopic expression of PU.1 increases IL-9 production. Reduced PU.1 expression in human IL-9-secreting T cell cultures also reduced IL-9 production. Mechanistic studies have shown that PU.1 likely influences T_H_9 differentiation by interfering with GATA3 activation or by recruiting the histone acetyltransferase (HAT) proteins Gcn5 and PCAF to the *Il9* locus [21, 38].

The TGFβ-activated kinase TAK1 is an important mediator of Smad-independent TGFβ signaling [50] and plays a key role in directing T_H_9 differentiation [33]. Our recent studies confirm that TAK1 inhibition reversed SIRT1 suppression, suggesting that a Smad-independent TAK1 signal is responsible for SIRT1 suppression during T_H_9 differentiation. SIRT1 deficiencies induced by either conditional deletion in mouse CD4^+^T cells or small interfering RNA (siRNA) in mouse or human T cells increased, while ectopic SIRT1 expression inhibited, IL-9 production. Additionally, glycolytic activation through the mTOR-hypoxia-inducible factor-1α (HIF1α) pathway was required for T_H_9 cell differentiation. SIRT1 may therefore function as a gatekeeper of the downstream mTOR-HIF1α axis (Figure 2). Furthermore, mTOR-HIF1α-IL-9 promoter transcriptional regulation coupled with modulation of glycolytic activity is selective for SIRT1-dependent T_H_9 cell differentiation [51].

Transcriptional factors downstream of IL-2 are critical for T_H_9 cell differentiation [24], and IL-2 deficient CD4^+^T cells do not produce IL-9. STAT5, a downstream target of IL-2, directly binds to the *Il9* locus and thus promotes T_H_9 cell differentiation. Mechanistic studies suggest that IL-2-STAT5 signaling inhibits B cell lymphoma 6 (Bcl6) expressions and T_H_17 cell generation, thereby promoting T_H_9 cell differentiation [24, 42].

The transcription factors NF-kB and NFAT also modulate T_H_9 cell differentiation. Ligation of OX40 triggers sustained activation of the non-canonical NF-kB pathway in CD4^+^T cells during T_H_9 cell differentiation [35, 36]. The non-canonical transcription factor NF-kB (RelB) directly binds to the *Il9* promoter region and triggers *Il9* transcription under T_H_9-inducing conditions. The non-canonical alternative NF-kB pathway probably also acts together with other factors to promote T_H_9 differentiation, suggesting that it restricts the capacity of NF-kB to interact with other transcription factors at the *Il9* locus. NFAT1 (nuclear factor of activated T cells) is also required together with NF-kB for IL-9 production in CD4^+^T cells [52]. NFAT1 alters histone modifications and chromatin structure and restricts RelA access to the *Il9* promoter region.

Transcription factors control the secretion of specific cytokines that direct T cell differentiation and differentiating T cells integrate multiple, and sometimes conflicting, signals as they differentiate into particular subsets. This is especially true for T_H_9 cell differentiation, during which cells integrate the T_reg_-inducing TGF-β signal and the T_H_2-inducing IL-4 signal to develop a specific T_H_9 cell phenotype. TGF-β signaling induces Foxp3, which is a negative regulator of T_H_9 cell differentiation; ectopic Foxp3 expression reduces IL-9 production in T_H_9 cells [35, 46]. The IL-4 signal targets multiple genes and induces expression of the transcriptional factors IRF4, GATA3, and STAT6. IRF4 is essential for T_H_2 and T_H_17, but not T_H_9, cell differentiation. GATA3 and STAT6 are also expressed in T_H_2 cells. Thus, each of these transcriptional factors likely plays integral roles in *Il9* gene expression and production in CD4^+^T cells. Therefore, these transcription factors may not directly involved in the transcriptional regulation of the *Il9* gene, but act rather as molecules downregulating negative factors during T_H_9 differentiation, such as Foxp3, or upregulating positive factors, such as IRF4, GATA3 and STAT6, during T_H_9 cell differentiation.

## T_H_9 CELL DIFFERENTIATION AND FUNCTION IN VARIOUS DISEASES

**Figure 3:**
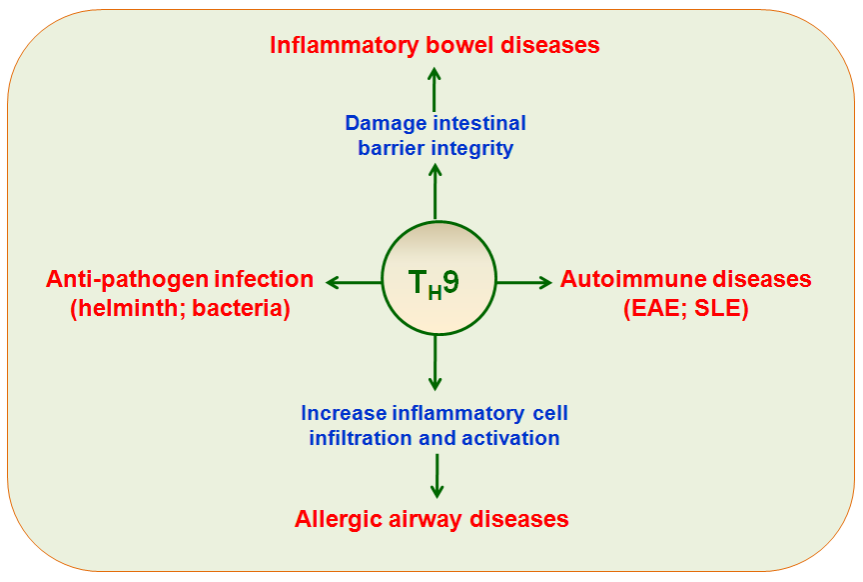
T_H_9 cells in immune-mediated diseases T_H_9 cells have potent protective or pathological roles in immune-mediated diseases, including allergic airway disease, inflammatory bowel diseases, autoimmune diseases (EAE, SLE), and pathogen infections.

**Figure 4:**
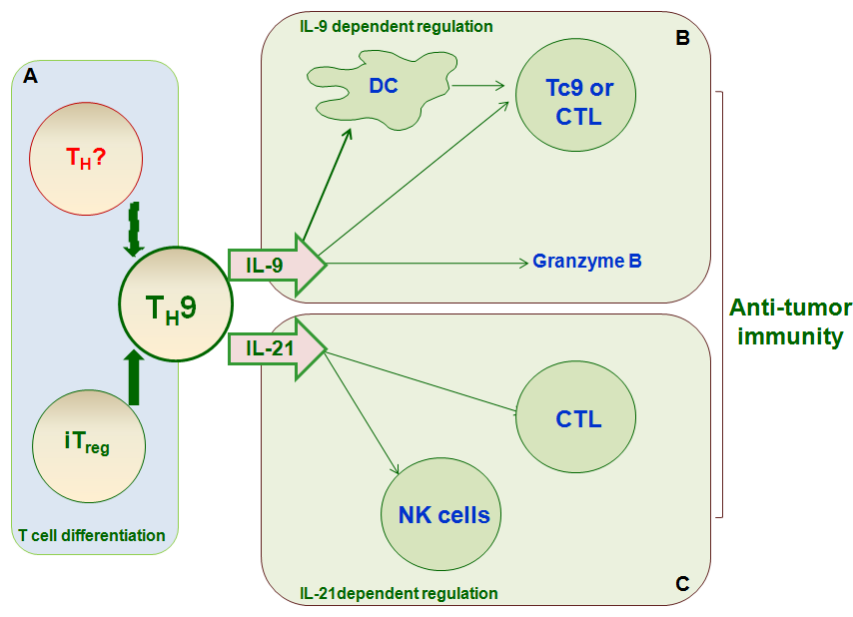
T_**H**_9 differentiation and function in anti-tumor immunity T_H_9 cells have anti-tumor activity, particularly in melanoma. **A.**, iT_reg_ cells and other T cell subsets can differentiate into T_H_9 cells, which affect anti-tumor immunity. T_H_9 cells exert anti-tumor effects primarily *via* the secretion of the cytokines IL-9 **B.** and IL-21 **C.** IL-9 promotes tumor cell death directly by increasing granzyme B release and indirectly by increasing DC survival and recruitment, inducing host anti-tumor CD8^+^CTL response, or increasing Tc9 activity. IL-21 contributes to anti-tumor immunity by promoting NK cell cytolytic function and CD8^+^CTL response.

T_H_9 cells have protective or pathological roles in several clinical diseases, including allergic diseases, autoimmune diseases, ulcerative colitis, infection with various pathogens, and anti-tumor immunity (Figure 3 and 4).

### T_H_9 cells in allergic airway diseases

Allergic airway diseases are characterized by T_H_2-associated cytokine responses to inhaled allergens, which are orchestrated by CD4^+^T cells that produce eosinophilic lung inflammation [14]. In addition to the T_H_2-associated cytokines IL-4, IL-5, and IL-13, the T_H_9-associated cytokines IL-9, IL-10, and IL-21 are increased in various diseases. The T_H_9-associated genes IL-4RA, STAT6, TGFβRII, PU.1, OX40, IL-9, IL-9R, SMAD3, and IL-33 are related to asthma [53–55]. Airway responses and inflammatory cell levels are often evaluated in OVA-sensitized mice in asthma research [56]. Such studies indicate that T_H_9 cells are the primary source of IL-9 in allergic airway diseases. Additionally, increased numbers of T_H_9 cells in draining lymph nodes (DLNs) and airways strongly correlate with allergic airway diseases. T_H_9 cell-derived IL-9 can exacerbate disease conditions by increasing inflammatory cell infiltration and activation within the respiratory tract. Numbers of circulating CD4^+^T cells and IL-9 secretion are increased in serum from allergic lung disease patients compared to controls [57, 58]. Importantly, adoptive T_H_9 cell transfers increased the development of allergic airway diseases following OVA challenge, while anti-IL-9 treatment reduced disease severity [34, 58–60]. These data suggest that T_H_9 cells are critical for the induction of allergic airway disease. Similarly, our work showed that severe pathogenic lung inflammation and inflammatory cell recruitment, including eosinophil infiltration in bronchoalveolar lavage fluid (BALF), were increased in SIRT1-deficient mice compared to WT mice. Additionally, more of the CD4^+^T cells isolated from lung DLNs of SIRT1-deficient mice were IL-9^+^. Importantly, administration of an anti-IL-9 antibody reversed pathological lung tissue damage and infiltration of inflammatory cells, including eosinophils and IL-9^+^CD4^+^T cells in SIRT1-deficient mice. Thus, SIRT1 is required for the suppression of T_H_9 cell differentiation and alleviates T_H_9-associated allergic airway inflammation [51].

T_H_2 and T_H_9 cells may play different roles in allergic airway diseases. We propose that, during the acute inflammation phase, activated T_H_2 cells secrete multiple cytokines (e.g. IL-4, IL-13). Subsequently, various cytokines promote mast cell recruitment (IL-4, IL-9, and IL-13), eosinophil maturation (IL-5 and GM-CSF), basophil infiltration (IL-4), and the initiation of mucus metaplasia (IL-13). During the chronic phase of inflammation, repeated exposure to allergenic antigens and damaged epithelium promotes the secretion of various cytokine growth factors, including TGFβ, from epithelial cells, eosinophils, macrophages, and even mast cells. In the presence of TGFβ and IL-4, T_H_2 cells differentiate into T_H_9 cells, which promote the progression of allergic airway diseases [56, 61, 62].

### T_H_9 cells in inflammatory bowel diseases

Inflammatory bowel diseases (IBD), which include Crohn's diseases (CD) and ulcerative colitis (UC), are characterized by sustained inflammation, mucosal barrier defects, and frequent intestinal infections. T_H_17 and iT_reg_ cells have been implicated in the pathological mechanisms of IBD. However, studies have also shown that T_H_9 cells are involved in IBD [63–65]. For example, adoptive transfer of T_H_9 cells into lymphopenic recipient mice (*Rag1*^−/−^; T and B cell-deficient mice) aggravated colitis, but the underlying mechanisms remain unknown. T_H_9 cells contribute to the pathogenesis of IBD, particularly UC, by regulating intestinal barrier integrity and immunological function [21, 66, 67]. Furthermore, IL-9 expression is elevated in patients with active UC and is highest in patients with the most severe disease. Patients with active UC also have more intestinal CD4^+^PU.1^+^T cells than control patients or patients with CD, indicating that T_H_9 cells are at least partly responsible for IBD pathogenesis. In acute and chronic IBD mouse models, numbers of IL-9-producing cells and IL-9R levels increased during colitis. In a novel IL-9 reporter mouse, CD4^+^T cells, but not other cell types such as innate lymphoid cells, are the principal source of IL-9 in colitis. Importantly, treatment with an IL-9 antibody or IL-9 knockout in mice considerably reduces colitis, as indicated by weight loss, generation of reactive oxygen species, and clinical scores, indicating that IL-9 promotes the progression of colitis. Thus, targeting IL-9 activity may improve diagnosis and treatment in UC patients.

### T_H_9 cells in autoimmune diseases

T_H_9 cells have been implicated in conditions that involve central nervous system inflammation, such as systemic lupus erythematosus (SLE), experimental autoimmune encephalitis (EAE), and systemic sclerosis (SSc) [19]. Adoptive transfer of MBP (myelin basic protein)-specific TCR transgenic T_H_9 cells into *Rag1*^−/−^ mice result in more severe EAE than transfer of T_H_1 or regular T_H_9 cells, indicating that T_H_9 cells play a critical role in the development of EAE [19, 68–70]. Due to complex regulatory mechanisms and variation in IL-9R singling in different immune cells during EAE, some studies have obtained conflicting results regarding the effects of IL-9 signaling in EAE [18, 43, 71]. IL-9^−/−^ mice develop less severe EAE than their WT counterparts following either immunization with myelin proteolipid protein (PLP; 180-199) peptide in the presence of Complete Freund's Adjuvant (CFA) or adoptive transfer of PLP (180-199) peptide-specific effector T cells from WT littermates. EAE-resistant IL-9^−/−^ mice exhibited considerably fewer infiltrating immune cells in the CNS, as well as reduced IL-17 and IFNγ expression. IL-9 deficiency also reduced PLP peptide-specific IL-17 and IFNγ levels [72, 73]. In addition, IL-9 receptor deficiency and IL-9 neutralization attenuated EAE and correlated with decreased numbers of T_H_17 cells and IL-6-producing macrophages in the central nervous system, and with decreased numbers of mast cells in regional lymph nodes [74]. However, some studies found that IL-9R^−/−^ EAE mice had more T_H_1 cells and T_H_17 cells than WT mice. Additionally, the inhibitory activity of T_reg_ cells was reduced in IL-9R^−/−^ mice compared to WT mice [71]. Together, these data suggest that IL-9 signaling may have a protective effect in EAE, although differential expression of IL-9 receptors in multiple cell types may result in more heterogeneous effects. However, the attenuation of EAE that results from IL-9 neutralization indicates that IL-9 signaling may also be pro-inflammatory.

To further investigate these possibilities, mRNA and serum IL-9 levels were assessed in the peripheral blood of SLE patients and healthy controls [75]. The percentage of CD4^+^IL-9^+^ T cells was elevated in SLE patients. Moreover, IL-9 expression in T cells and serum IL-9 levels in 8 untreated active SLE patients decreased 1, 2, and 3 weeks after treatment with methylprednisolone [75]. Thus, T_H_9 plays an important role in the pathogenesis of SLE.

### T_H_9 cells in anti-pathogen infection activity

IL-9 production in T_H_9 cells also results in anti-helminth activity. Experiments in IL-9^−/−^ mice and IL-9-fluorescent reporter mice indicate that IL-9 is crucial in the initiation of host-protective responses in the early type 2 immune response against *Nippostrongylusbrasiliensis* [15]. Adoptive transfer of T_H_9 cells, but not T_H_2 cells, caused rapid worm expulsion and marked basophilia and increased mast cell numbers in *Rag2*^−/−^ mice [15]. T_H_9 cells and IL-9 are also involved in human *Echinococcusgranulosus* infection [76]. Compared to healthy controls, PU.1, IL-9, and GATA-3 mRNA expression were increased in untreated patients. In addition, an increase in T_H_9 cells with the effector memory cell phenotype was found in tuberculous pleural effusion [16]. Taken together, these data suggest that T_H_9 cells and IL-9 a play critical and non-redundant roles in host-protective immunity against pathogenic infection.

## T_H_9 CELL DIFFERENTIATION AND FUNCTION IN ANTI-TUMOR IMMUNITY

### T_H_9 cells regulate tumor immunity

T_H_9 cells are present in metastatic pleural effusion [77] and human melanoma tumor-infiltrating lymphocytes [78]. T_H_9 also has anti-tumor properties in mice [77, 79] (Figure 4). In addition, melanoma tumor growth is accelerated in IL-9R^−/−^ mice, and treatment with rIL-9 inhibited this growth. Interestingly, adoptive transfer of anti-tumor T_H_9 cells inhibited tumor growth, and administration of an anti-IL-9 mAb reversed this effect. Moreover, treatment with exogenous IL-9 suppressed the growth of B16F10 melanoma and LLC-1, but not EL-4, tumors. Mechanistic investigation revealed that IL-9R expression is negligible in B16F10 and LLC-1 cells, and IL-9 had minimal effects on growth in these cells [80, 81]. However, IL-9R expression is increased in EL-4 cells, indicating an additional effect of IL-9 on tumor cells; evaluation of IL-9R expression may therefore be critical for IL-9 anti-tumor therapy. These results have been confirmed [77] and show that adoptive transfer of antigen-specific T_H_9 cells exerts anti-tumor effects by inhibiting the subcutaneous lung metastasis of B16F10 and T_H_9 cells. In addition, T_reg_, T_H_17, and T_H_2 cells also secrete low levels of IL-9. A considerable portion of T_H_9 cells acquire the T_H_1 phenotype and produce IFN-γ *in vivo* [82, 83]. T_H_9 cell plasticity therefore plays a role in various pathological processes, including cancer.

Our studies also indicate that T_H_9 cells contribute to anti-tumor immunity. Naïve T cells isolated from WT or SIRT1-deficient mice were differentiated under T_H_9-inducing conditions and transferred into *Rag*1^−/−^ mice, which were then subcutaneously injected with B16 melanoma cells. The mice that received SIRT1-deficient CD4^+^T cells developed smaller tumors than WT controls. Tumor infiltrating CD4^+^T cells isolated from the SIRT1-deficient groups had a higher IL-9^+^ ratio than the WT group. Importantly, administration of an anti-IL-9 antibody reversed the changes induced by SIRT1-deficient T_H_9 cell transfer [51]. These data show that SIRT1 is required for the suppression of T_H_9 differentiation, and thus inhibits T_H_9-dependent anti-tumor immunity.

### Regulatory mechanisms of T_H_9 cells in anti-tumor immunity

IL-9 produced by T_H_9 cells elicits anti-tumor immune responses through both direct and indirect regulatory mechanisms.

Several studies have reported direct regulatory mechanisms of T_H_9 cells. For example, IL-9 produced by CD4^+^TCells inhibits melanoma HTB-72 cell growth by upregulating p21 and TRAIL [84]. Most blood- and tissue-derived human memory T_H_9 cells were skin-tropic or skin-resident and co-expressed TNFα and granzyme B, suggesting that they play a pro-inflammatory role [85]. Two recent studies [86, 87] also demonstrated that glucocorticoid-induced TNF receptor-related protein (GITR) ligation directs the differentiation of iT_reg_ cells into T_H_9 cells and thus mediates anti-tumor immunity, indicating that reciprocal differentiation of T cell lineages is critical for the anti-tumor effects of T_H_9 cells. Cca cell extract (CT26 cells) acts as an antigen and induces T_H_2 responses in Cca-bearing mice, thus inhibiting tumor growth primarily by converting intra-Cca T_reg_s cells into T_H_9 cells [12]. Finally, T_H_9 cells have stronger anti-tumor effects than T_H_17 or T_H_1 cells [22], but the reciprocal differentiation mechanisms in these T cell lineages and their effects on tumor response require further investigation.

Indirect regulatory mechanisms of T_H_9 cells are likely also critical for anti-tumor immunity. IL-9 produced by CD4^+^T cells is responsible for increased survival and function in myeloid DCs, which contribute to anti-tumor immunity [88]. T_H_9 cells also induce host anti-tumor CD8^+^CTL responses by upregulating the CCL20/CCR6-dependent recruitment of dendritic cells (DCs) to local tumor tissues [77]. These findings suggest that T_H_9-mediated immune regulation is crucial for anti-tumor immunity. Moreover, tumor-specific IL-9-producing CD8^+^Tc9 cells also exert potent anti-tumor effects [89]. Tc9 cells primed by T_H_9-inducing conditions secreted different cytokines and were less cytolytic *in vitro*, but exhibited much stronger anti-tumor effects in OT-I/B16-OVA and Pmel-1/B16 melanoma mouse models. Adoptive transfer of Tc9 cells results in differentiation into IFNγ- and granzyme-B (GrzB)-producing cytolytic Tc1-like effector cells. This suggests that the anti-tumor activities of T_H_9 cells are partially due to their effects on Tc9 cells.

In addition to IL-9, IL-21 secreted by T_H_9 cells also exerts critical anti-tumor effects. IL-1β promotes the secretion of cytokines IL-9, IL-10, and IL-21 from T_H_9 cells through STAT1-IRF1-dependent mechanisms [22, 90, 91]. Furthermore, this IL-1β-induced T_H_9 differentiation increased IL-21, but not IL-9, secretion, and increased IL-21-dependent anti-tumor effects [92–94]. IL-21 stimulates IFNγ production, enhances the cytolytic activity of NK cells, and increases CD8^+^CTL activity, all of which promote anti-tumor immunity [28, 94].

## CONCLUDING REMARKS

Following antigen stimulation, naïve CD4^+^T cells differentiate into one of several functional effector cell classes. In addition to the classical T_H_1 and T_H_2 lineages, T_H_17 cells have been extensively characterized. Recently, new subsets of IL-9-producing CD4^+^T cells have been induced *in vitro* by IL-4 and TGFβ and identified *in vivo*. IL-9 is a pleiotropic cytokine produced by T_H_2 cells, mast cells, and eosinophils. Due to the critical role of IL-9-producing CD4^+^T cells in immune-mediated diseases, T_H_9 cells have been extensively investigated in mouse and human studies during the past 20 years. These studies have demonstrated that T_H_9 cells contribute to both immune responses and immunopathological diseases. Moreover, they suggest that promoting or suppressing T_H_9 cell differentiation, transcriptional control, and function may provide novel treatments for T_H_9-associated inflammation, autoimmune diseases, and tumors. Specifically, administration of IL-9 antibodies effectively eliminated T_H_9 cells and ameliorated or aggravatedT_H_9-associated diseases.

## ABBREVIATIONS

BCL6: B cell lymphoma 6
CD: Crohn's diseases
DCs: dendritic cells
EAE: experimental autoimmune encephalitis
IL-9: interleukin 9
IBD: Inflammatory bowel diseases
iT_reg_ cells: inducible regulatory T cells
HAT: histone acetyltransferase
NFAT: nuclear factor of activated T cells
NK cells: natural killing cells
NF-kB: nuclear factor-kappa B
NICD: notch intracellular domain
RBP-Jk: recombination signal binding protein for immunoglobulin kappa J region
STAT6: signal transducer and activator of transcription 6
SLE: systemic lupus erythematosus
SSc: systemic sclerosis
TNFα: tumor necrosis factorα
TSLP: thymic stromal lymphopoietin
TGFβ1: transforming growth factor β1
T_H_9: IL-9 producing CD4^+^T cells
GITR: TNF receptor-related protein
GrzB: granzyme-B
UC: ulcerative colitis
